# Helicopter emergency medical services use of thoracic point of care ultrasound for pneumothorax: a systematic review and meta-analysis

**DOI:** 10.1186/s13049-021-00977-0

**Published:** 2021-11-20

**Authors:** Edward Griffiths

**Affiliations:** 1 Bristow Helicopters Search and Rescue, UK Search and Rescue Helicopter Service, Aberdeen, UK; 2 Queen Mary University London, London, UK

**Keywords:** HEMS, Pre-hospital, Pneumothorax, Ultrasound, POCUS

## Abstract

**Background:**

Auscultating for breath sounds to assess for pneumothorax in the helicopter emergency medical services (HEMS) settings can be extremely challenging. Thoracic point of care ultrasound (POCUS) offers a seemingly more useful visual (rather than audible) alternative. This review critically and quantitatively evaluates the use of thoracic POCUS for pneumothorax in the HEMS setting.

**Methods:**

A systematic literature review with meta-analysis was conducted. Only papers reporting on patients undergoing POCUS for pneumothorax in the helicopter or pre-hospital setting were included. Primary outcome was accuracy, focusing on sensitivity and specificity. Secondary outcome was practicality. PubMed, Embase and the Cochrane Library were searched. The Quality Assessment of Diagnostic Accuracy Studies (QUADAS-2) was used to assess validity of studies.

**Results:**

Twelve studies reporting on *n* = 1,936 images from medical and trauma patients were included in qualitative synthesis. Studies were nearly all observational designs. Most images were acquired by nurses or paramedics who were previously novices to ultrasound. The reference standard was predominantly CT. Specificity results were unanimously precise and very high, whereas sensitivity results were imprecise and extremely variable. Meta-analysis of eight studies involving *n* = 1,713 images yielded pooled sensitivity 61% (95% CI: 27–87%; *I*^2^ = 94%) and pooled specificity 99% (95% CI: 98–100%; *I*^2^ = 89%). Six studies involving *n* = 315 images reported practicality. The highest or second highest categorisation of image quality was reported in around half of those images.

**Conclusion:**

Thoracic POCUS is highly specific but has extremely variable sensitivity for pneumothorax when performed in the HEMS setting. This is from purely a diagnostic (not clinical) perspective. Sensitivity increases when only clinically significant pneumothoraces are considered. Case reports reveal thoracic POCUS can appropriately alter treatment and triage decisions, but only for a small number of patients. It appears predominantly useful in mitigating against unnecessary interventions. More research reporting patient focused outcomes is required. In the meantime, thoracic POCUS appears to offer a more appropriate visual alternative to auscultation for breath sounds when assessing for pneumothorax in the HEMS setting.

## Introduction

### Rationale

Helicopter emergency medical services (HEMS) provide pre-hospital critical care and interfacility transfers. They encounter patients presenting with pneumothorax and tension pneumothorax. Pneumothorax occurs when air enters the pleural cavity through a plural fault. These faults may have traumatic, idiopathic (spontaneous or relating to disease) or iatrogenic (related to medical intervention) causes. Prevalence of pneumothorax amongst patients presenting to HEMS providers is reported as being between 10 and 20% [1–6].

Tension pneumothorax occurs when the plural fault functions as a one-way valve [7]. Air continues to enter the plural cavity more quickly than it can escape. An increase in intrapleural pressure ensues [7]. This causes lung collapse, diaphragmatic depression, chest wall expansion and contralateral lung compression [7]. Eventually, compression of the thoracic vena cava ensues [7]. This leads to reduced venous return and eventual circulatory collapse [7]. It is an immediate life-threatening condition portrayed by these clinical manifestations. Hence, early recognition and immediate treatment are imperative. Similarly, timely identification of a simple pneumothorax alerts clinicians to the risk of inducing a tension pneumothorax in patients who undergo positive pressure ventilation or altitude related volume expansion [8].

One of the cornerstones of pre-hospital assessment for pneumothorax is auscultation of the chest. However, auscultating to determine the presence of breath sounds in the pre-hospital setting can be extremely challenging. Brown et al. evaluated the accuracy of auscultation by pre-hospital clinicians to detect breath sounds whilst in a moving ambulance [9]. In a sample of *n* = 260, they reported *n* = 117 false negatives [9]. Similarly, Hunt et al. concluded that auscultation for breath sounds in the helicopter environment was impossible [10]. The inability to auscultate in this setting renders differentiating pneumothorax seemingly more difficult. There appears greater potential for pneumothorax going undiagnosed.

A recent meta-analysis reported a 19% complication rate associated with performing a thoracostomy [11]. These included iatrogenic injury, bleeding, and infection. Hence, there also appears a risk of clinicians unnecessarily exposing patients to the risk of these complications due apparent greater difficulties in ruling out a pneumothorax in the HEMS setting [12].

### Clinical role of index test

**Fig. 1:**
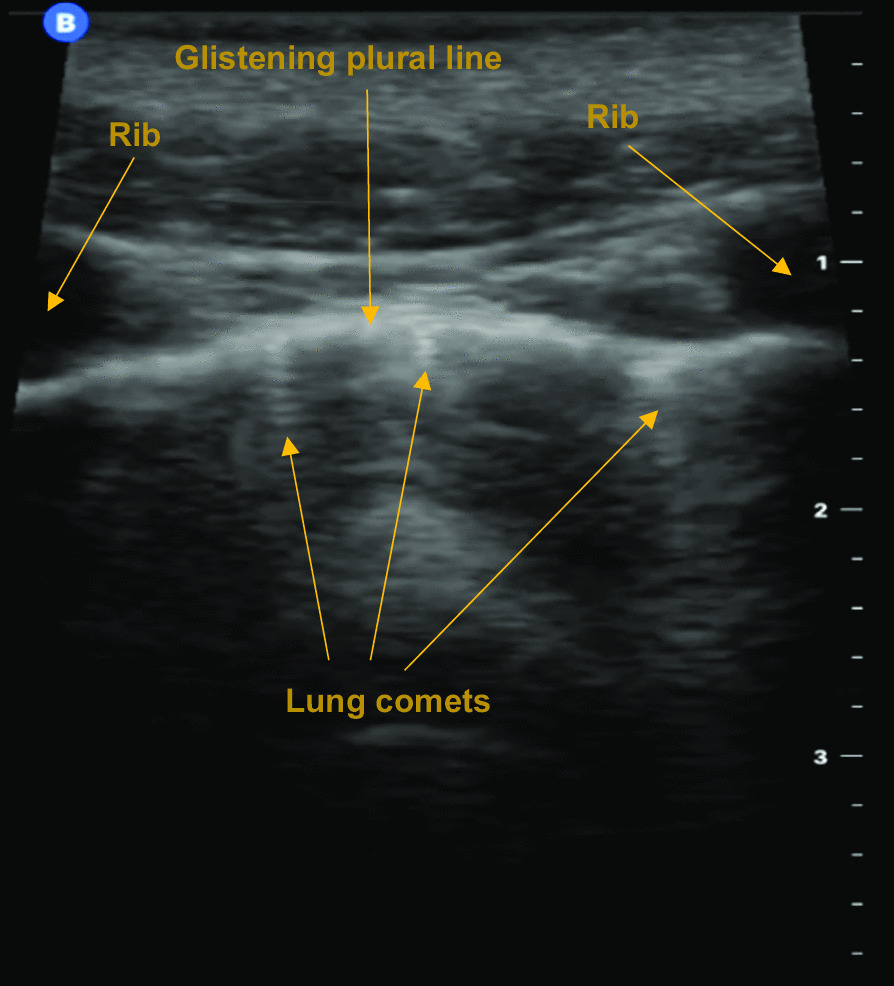
Lung sliding (glistening plural line) accompanied by lung comets

**Fig. 2:**
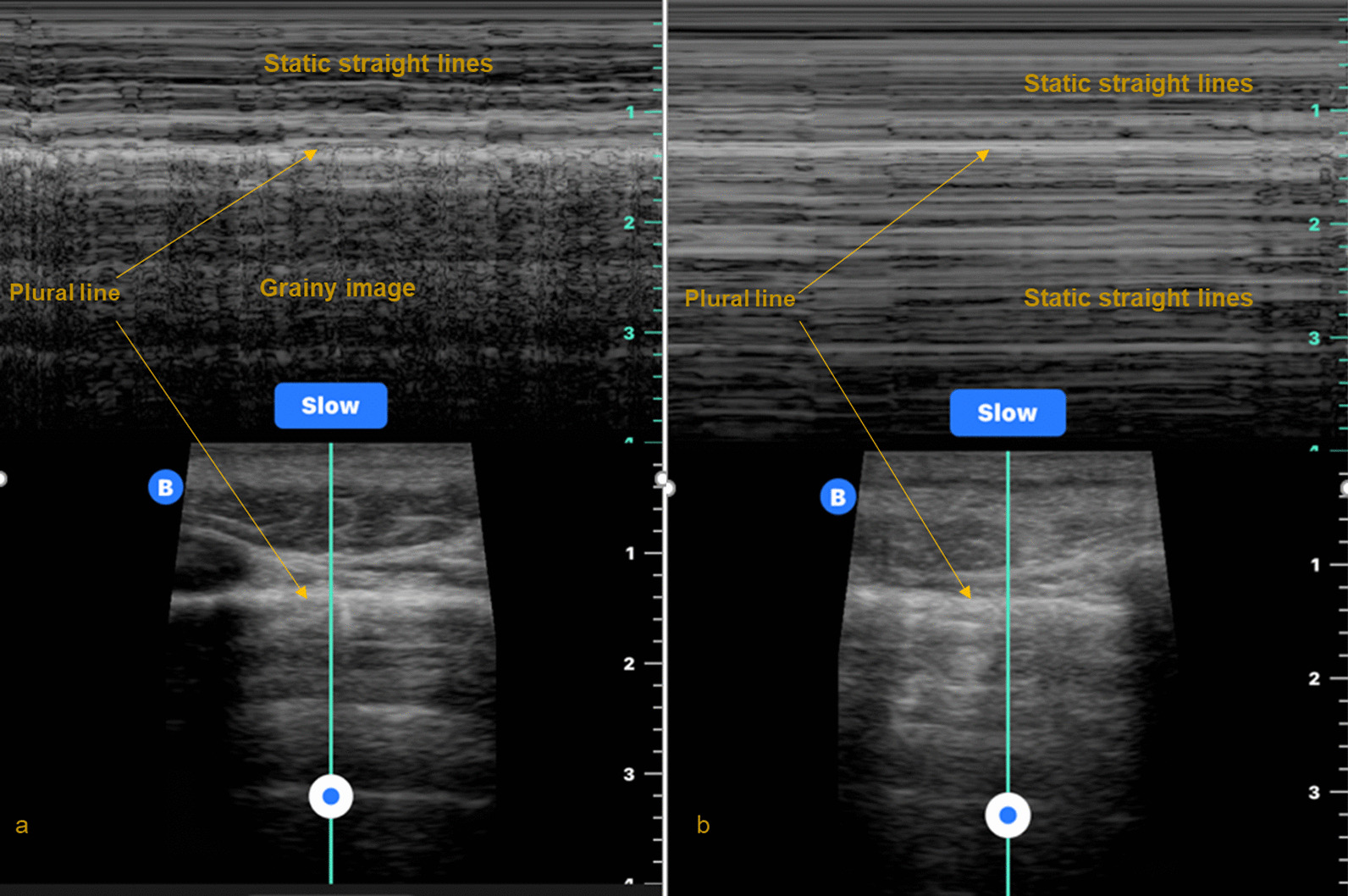
**a** The seashore sign depicting lung sliding. **b** The barcode (or stratosphere) sign associated with absence of lung sliding

The advent of handheld ultrasound machines combined with their improved image quality has brought a point of care ultrasound (POCUS) capability into the HEMS arena. Ultrasonic imaging of underlying anatomy can now be depicted on handheld electronic tablets or smart phone devices. They can depict the visceral pleura sliding on the parietal pleura (termed *lung sliding*) as a glistening movement at the plural line (Fig. 1) [13, 14]. In motion mode (*M-mode*), movement of the lung appears as a grainy image below the plural line, while the still chest wall above is depicted as static straight lines. This is termed the *seashore sign *(Fig. 2a) [13, 14]. Pneumothorax pathology results in an absence of lung sliding and the resultant M-mode image is that of parallel horizontal lines above and below the pleural line (Fig. 2b) [13, 14]. This pattern is often referred to as the barcode or stratosphere sign (Fig. 2b) [13, 14].Thoracic POCUS can also depict *lung pulse* (pulsation of the heart transmitted through lung tissue) which is present in normal lung, but absent in pneumothorax [13, 14].

Another useful observation when assessing for pneumothorax are *lung comets*. These are caused by reverberation of ultrasound waves at the peripheral lung parenchyma and inter-plural layer (Fig. 1) [15]. They appear as short (typically less than 1 cm) vertical artefacts beginning at the plural line, which then taper and fade with increasing depth (Fig. 1) [15]. Fibrosed lung interstitium or a mixing of air and fluid in the interstitium can cause another phenomenon termed *B-lines* [14, 15]. These also appear as bright vertical lines, but are much longer than lung comets—they shine down from the pleura to the end of the screen [15]*.* Lung comets and B-lines move with lung sliding [15]; as they both arise from lung tissue and/or the inter-plural layer, their presence can be used to discount pneumothorax [14]. Lung comets are the more useful diagnostic observation as they are ubiquitous irrespective of disease status [15].

By virtue of its visual modality, thoracic POCUS appears to offer a superior alternative to auscultation of the chest to aid diagnosis of pneumothorax in the HEMS setting.

### Objectives

Recent updates to resuscitation guidelines place greater emphasis on the use of POCUS to identify underlying pathology and target resuscitative interventions [16, 17]. They make specific mention of the merits of its use for assessing for pneumothorax [16, 17]. However, no quantitative analysis focusing on the pre-hospital or helicopter setting has been published to date. The aim of this paper was to addresses this evidentiary gap by conducting a systematic review and meta-analysis of the accuracy and practicality of thoracic POCUS for pneumothorax in the HEMS setting. Measurements of accuracy focussed on sensitivity and specificity. Practicality was measured as declared rates of diagnostically adequate images, or practicality rating.

## Methods

All elements of the Preferred Reporting Items for Systematic Reviews and Meta-Analysis for Diagnostic Test Accuracy (PRISMA-DTA) studies checklist are reported under separate subheadings [18].

### Protocol and registration

The protocol for this review was prospectively submitted to the International Prospective Register of Systematic Reviews (PROSPERO) on the 21st of November 2020. It was first published on the PROSPERO database on the 2nd of December 2020 (Registration No. CRD42020221946).

### Eligibility criteria

To be included for analysis, results had to meet all the following *population*, *index-test*, *reference test* and *target condition* eligibility criteria:

1. Population—all patients being treated in the helicopter or pre-hospital setting.
2. Index test—thoracic POCUS.
3. Reference test—computed tomography (CT), X-ray, subsequent expert interpretation of saved images or operative/clinical findings.
4. Target condition—reporting on accuracy or practicality in identifying the presence or absence of pneumothorax.

Only randomised trials and non-randomised studies were eligible for inclusion. Articles which were not available in full text or not written in English were excluded.

### Information sources

PubMed (includes MEDLINE), Embase and the Cochrane Library were searched between 4th and 15th January 2021. An additional search (guided by GreyNet.org) for papers not published in mainstream journals was also conducted. Reference lists of the search results were checked for studies which were eligible for inclusion. A search of the International Clinical Trials Registry Platform (which includes ClinicalTrials.gov) was also completed.

### Search

*MeSH* and *EMTREE* terms were customised to search PubMed (also adopted by the Cochrane Library) and Embase respectively. A free-text search for key terms (including their synonyms and related terms) appearing in titles and abstracts was also conducted. Searches using *MeSH* and *EMTREE* terms were ‘exploded’ to automatically search the respective subheadings where appropriate. Search terms are included at “Appendix 1”.

### Study selection

**Fig. 3:**
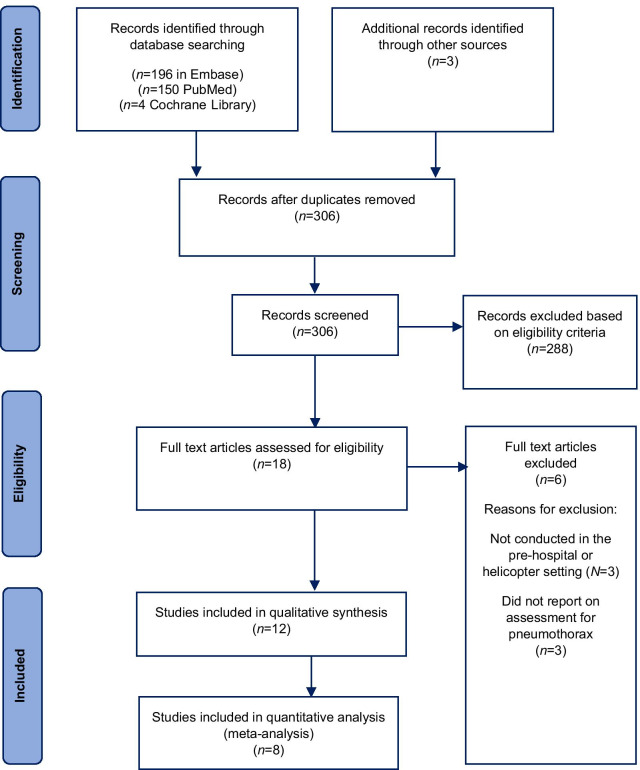
PRISMA flow diagram—study selection

The study selection process is depicted in Fig. 3.

### Data collection process

Data was extracted into a data collection table generated as a spreadsheet using Microsoft Excel (Version 16.0.13628.20274, Office 365, Microsoft Corporation, Redmond, Washington, 2021).

### Definitions for data extraction

Data items harvested for analysis are presented as column headings in Table 1.

**Table 1:** Characteristics of included studies

Study		Population				Index test (thoracic POCUS)			Reference test(s)	Funding sources
Authors	Design	Sample size (no. of images evaluated)	Setting	Presentation	Target condition	Operator(s)	Device	Method		
*Included in meta-analysis*										
Quick et al. [3]	Prospective observational	149	In-flight	Trauma (n = 136) and medical (n = 13)	Pneumothorax	Flight nurses and paramedics	Sonosite M-Turbo	Not specified	CT, X-ray, or clinical signs	None declared
Ronaldson et al. [5]	Observational	19	Ambulance (n = 5), helicopter (n = 3), roadside (n = 3), fixed wing (n = 1)	Trauma (50%) and medical (50%)	Pneumothorax	Flight nurses and paramedics	GE v-scan dual probe	2nd intercostal space	Expert review of original images	Not applicable
Yates et al. [6]	Prospective observational	190	On scene (35%), in-flight (51%), both (14%)	Trauma	Pneumothorax	Flight nurses and paramedics	Sonosite M-Turbo	eFAST	CT, or trauma surgeon’s operative report	None declared
Oliver et al. [1]	Retrospective observational	662	On scene, in ambulance, or in helicopter	Trauma	Pneumothorax	Physicians (66%)/ paramedics (34%)	GE v-scan dual probe and Sonosite i-Look	Not specified	CT	None declared
Press et al. [2]	Prospective observational	489	On scene and in-flight	Trauma	Pneumothorax	Flight nurses and paramedics	Sonosite M-Turbo	eFAST	CT, X-ray, or clinical signs	Sonosite, Inc
Ketelaars et al. [22]	Retrospective observational	59	On scene and in-flight	Trauma and cardiac arrest	Pneumothorax	Physicians	Sonosite MicroMaxx	PREP (comparable to eFAST)	CT	None declared
Roline et al. [4]	Prospective observational	81	In-flight	Medical and trauma	Pneumothorax	Not specified	Sonosite MicroMaxx	2nd intercostal space	Expert review of original images	None declared
Lyon et al. [24]	Prospective observational	64	In-flight (using a model to simulate plural interface)	Simulated	Pneumothorax	Not specified	GE Logique	M-mode imaging	Expert review of original images	None declared
*Not included in meta-analysis*										
Scharonow et al. [25]	Prospective observational	71	On scene	Medical (68%) and trauma (31.3%)	Pneumothorax	Physicians	Sonosite MicroMaxx	Pre-hospital lung ultrasound (PLUS) exanimation	CT, ultrasound (ED) or X-ray	No funding received
Neesse et al. [23]	Prospective observational	56	On scene	Medical	Pneumothorax	Physicians	Sonosite MicroMaxx	Anterior intercostal spaces two-to-four views	CT, ultrasound (ED) or X-ray	None declared
Khalil et al. [26]	RCT	60	Pre-hospital simulation	Trauma	Tension pneumothorax	Paramedics	SonoSim	Not specified	Pre-programmed simulated presentation of pneumothorax	None declared
Snaith et al. [33]	Prospective observational	36	ED versus stationary ambulance versus moving ambulance	Simulation—healthy subjects	Pneumothorax	Physicians and sonographers	Sonosite Nanomaxx	EFAST	Expert review of original images	Sponsored by the University of Bradford

### Risk of bias and applicability

The Quality Assessment of Diagnostic Accuracy Studies (QUADAS-2) assessment tool as described in a publication by Whiting et al. was used to assess the internal and external validity of studies [19]. Funnel plot asymmetry analysis for reviews of diagnostic studies developed by Deeks et al. was used to assess for the presence of publication bias [20].

### Diagnostic accuracy measures

STATA (StataCorp, Stata Statistical Software: Release 16. College Station, TX: StataCorp LLC, 2019) was used to calculate each study’s prevalence of pneumothorax, sensitivities, specificities, positive predictive value (PPV), negative predictive value (NPV) and the respective 95% confidence intervals (CI) using the data extracted. Review Manager (Version 5.4.1. Copenhagen: The Nordic Cochrane Centre, The Cochrane Collaboration, 2020) software was used to create forest plots of sensitivity and specificity.

### Synthesis of results

Practicality and accuracy were reported in qualitative thematic synthesis. The intention was to perform subgroup analysis to account for differences between the helicopter (in-flight) setting versus the pre-hospital.

### Meta-analysis

STATA statistics software package (StataCorp, Stata Statistical Software: Release 16. College Station, TX: StataCorp LLC, 2019) was used to conduct the meta-analysis. It focused on summarising sensitivities and specificities using a random effects model [21]. A positive or negative result (presence or absence of pneumothorax) was modelled as a single common binary threshold across all studies.

### Additional analyses

The intention was to conduct sensitivity analysis to account for the biases and concerns reported using the QUADS-2 tool. Practicality was reported using simple descriptive statistics. The intention was to present mean acquisition rates (percentages) of diagnostically adequate images.

## Results

### Study selection

Eighteen studies met the inclusion criteria [1–6, 22–33]. Full text reviews resulted in three studies being rejected as they did not involve image acquisition and interpretation in the pre-hospital or helicopter setting [27–29]. A further three were also rejected as they did not involve assessment for pneumothorax [30, 32, 34]. Of the remaining twelve studies included in qualitative synthesis [1–6, 22–26, 33], eight reported sufficient and appropriate data for inclusion in meta-analysis [1–6, 22, 24]. Figure 3 depicts this study selection process.

### Study characteristics

All twelve included studies were published between 2011 and 2020 [1–6, 22–26, 33]. Collectively, they reported on the interpretations of *n* = 1,936 images captured predominantly from trauma patients presenting in the pre-hospital and/or in-flight setting. All included patients were adults (≥ 18 years). Apart from one randomised simulation trial [26], all were observational designs. Results involved overall quantitative and qualitative evaluations, including the raw data used to make these calculations and subjective conclusions. Although the protocols of ultrasonography varied, they all included comparable elements of thoracic scanning to evaluate for pneumothorax. The majority (58%, *n* ≈ 1,132) of images were acquired by nurses or paramedics who had undertaken familiarisation training to enable them to participate in the studies. They were previously novices in thoracic ultrasound. Around 34% (*n* ≈ 659) were acquired by physicians experienced and accredited in ultrasound use, or by experienced sonographers. Authors did not declare the experience of those conducting the scans in around 8% (*n* ≈ 145) of images acquired. The reference standard was predominantly CT scanning. However, there were exceptions; these included X-ray, emergency department (ED) clinical assessment (including ultrasound) and expert review of the saved images. One study was funded by an academic institution [33], another was funded by the manufacturer of the ultrasound device used in the study [2]. The remaining studies either declared that no funding had been received, or they made no comment about sources of funding. Included study characteristics are summarised in Table 1.

### Risk of bias and applicability

**Fig. 4:**
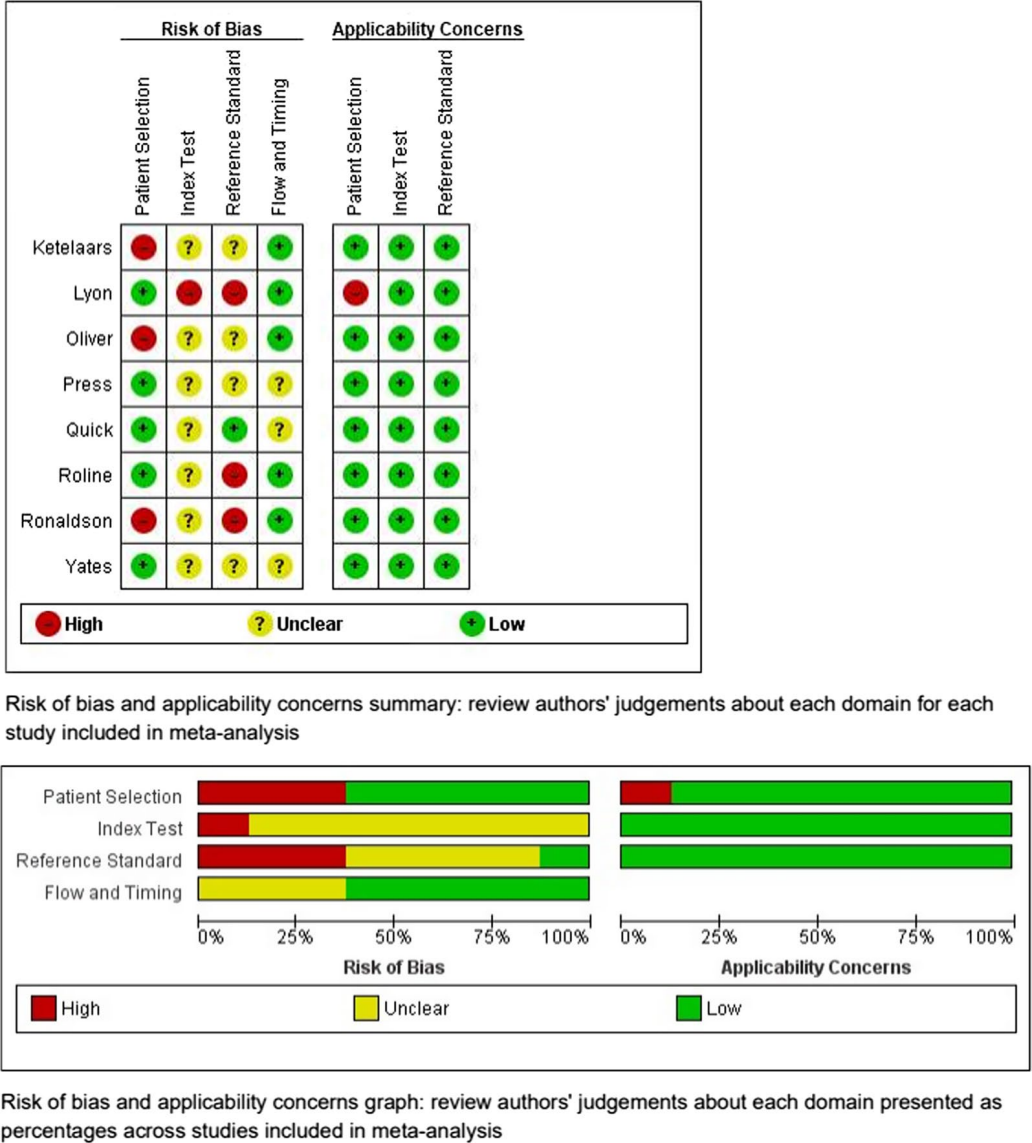
Risk of bias and applicability concerns for each study included in meta-analysis

Figure 4 depicts the risk of bias and applicability concerns of the eight studies included in meta-analysis [1–6, 22, 24]. Of these eight studies, five were assessed as having a high risk of bias in at least one domain and/or area of applicability. The remaining three studies involved an unclear risk of bias. Derivation of the risk of bias and applicability results are presented in “Appendix 2”.

## Results of individual studies: qualitative synthesis

### Accuracy

Eleven studies reported on the accuracy of a total of *n* = 1,900 separate images [1–6, 22–26]. The majority (60%, *n* ≈ 1,132) were acquired by paramedics or nurses; 32% (*n* ≈ 623) were acquired by physicians; the authors did not declare the credentials of the device operator for the remaining 8% (*n* = 145).

The Neesse et al. and Scharonow et al. studies involved patients undergoing thoracic POCUS examinations performed by physicians certified in sonography [23, 25]. Results were compared with CT, ED ultrasound or X-ray. Hospital staff were blinded to the results of pre-hospital imaging results. Pneumothorax was correctly ruled-out in all patients in both these studies. This zero prevalence can be explained by these studies reporting on predominantly non-trauma patients. Although these studies were of interest as they reported the apparent ability of thoracic POCUS to correctly rule out pneumothorax in a population of predominantly medical patients, they were excluded from meta-analysis on account of them reporting a zero prevalence of pneumothorax [23, 25]. Khalil et al. randomised *n* = 30 paramedics to undertake a 30-min cardiac and thoracic POCUS lecture followed by practical scanning of *n* = 10 volunteer subjects [26]. This intervention group was compared to *n* = 30 paramedics with no additional training, the majority of whom (*n* = 28, 93%) had never performed a POCUS examination. Both groups were then exposed to blinded simulation scenarios, one of which involved a tension pneumothorax in the pre-hospital setting. The simulation involved loud noise to hinder auscultation. Most paramedics (*n* = 27, 90%) in the intervention group utilised thoracic POCUS during their examination of the pneumothorax patient, whereas only *n* = 2 (7%) utilised it in the control group. Although a higher percentage of paramedics correctly diagnosed the tension pneumothorax in the intervention group (77% versus 57%), this difference was not considered statistically significant (*p* = 0.1). Although the sample size met the power calculation requirements, it relied on the premise that the thoracic POCUS education curriculum would improve diagnostic accuracy by 35%. No references were cited to substantiate modelling the required sample size on this magnitude of improvement.

**Fig. 5:**
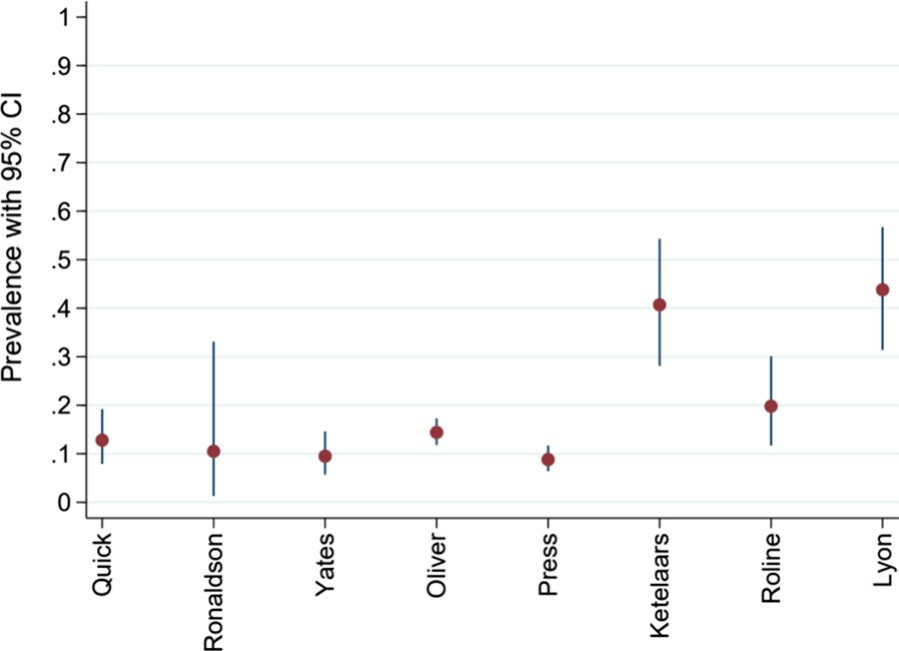
Prevalence of pneumothorax in each study included in meta-analysis

**Fig. 6:**
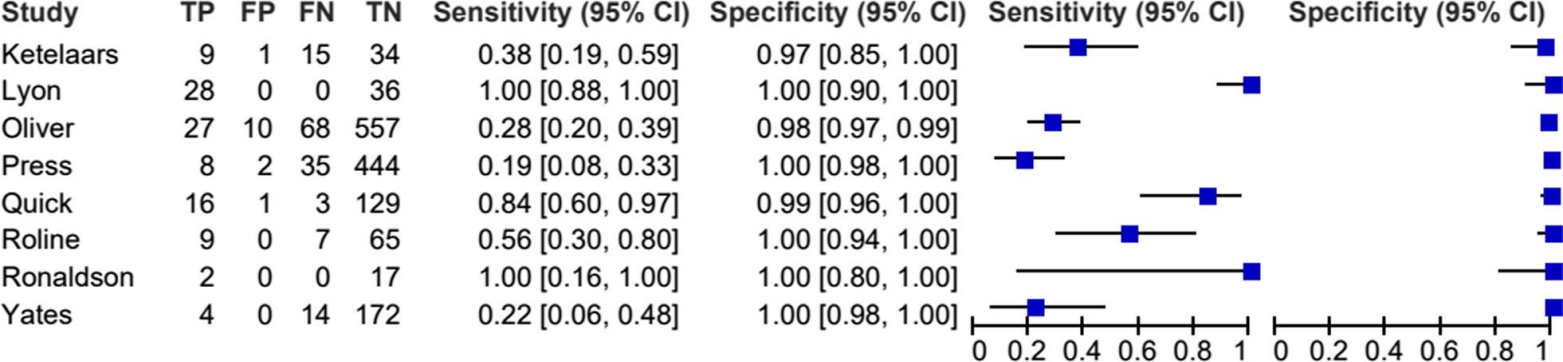
Forest plot of sensitivity and specificity of POCUS for pneumothorax in each study included in meta-analysis. Abbreviations: True positive (TP), false positive (FP), false negative (FN) and true negative (TN)

**Fig. 7:**
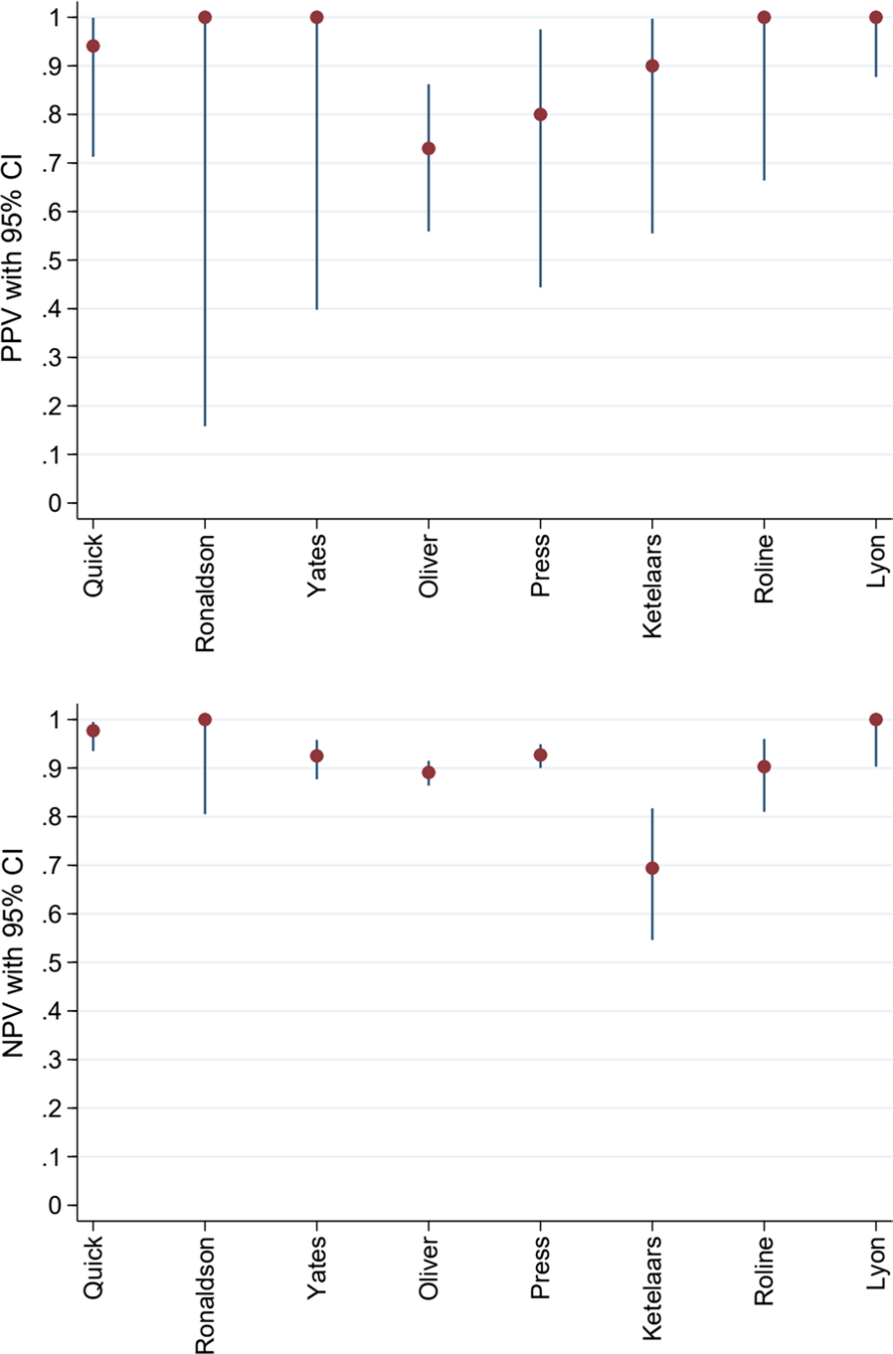
Predictive values of POCUS for pneumothorax in each study included in meta-analysis

The remaining *n* = 8 studies reporting accuracy data were included in the meta-analysis [1–6, 22, 24]. This data is summarised in Figs. 5, 6 and 7. Pertinent additional aspects of these studies are described in more detail below.

Prevalence of pneumothorax in the eight studies included in the meta-analysis was between 10 and 20% (Fig. 5) [1–6, 22, 24]. Exceptions were Ketelaars et al. who reported a prevalence of 40% (95% CI: 28–55%) and Lyon et al. who simulated a prevalence of 44% (95% CI: 31–57%) [22, 24]. PPV results were typically high but blighted by poor precision. This was on account of the relatively few images analysed. NPVs were similarly high but more precise (Fig. 7). In the Quick et al. study, accuracy reduced to sensitivity 68% (95% CI: 46–85%) and specificity 96% (95% CI: 90–98%) when the sample was limited to only patients who underwent CT as the reference test (*n* = 116) [3]. The authors stressed that all those that did not undergo CT had clear signs of pneumothorax on X-ray, or definitive clinical signs.

Multivariate binomial regression analysis in the Oliver et al. study revealed none of the variables observed had a significant effect on accuracy [1]. These included the operator’s clinical discipline (paramedic or physician), time from POCUS examination to CT scan, means of transportation (ground versus air), patient demographic and mechanism of injury [1].

### Practicality

Six studies rated the quality of a total of *n* = 315 separate images [4, 5, 22–24, 33]. Most (48%, *n* = 151) were acquired by physicians, 6% (*n* = 19) were acquired by non-physicians; authors did not declare the credentials of the device operator for the remaining 46% (*n* = 145). Methods of categorising image quality varied greatly between studies. Images in the Ketelaars et al. study were evaluated as *Good* (55%), *Moderate* (25%), *Poor* (14%) and *Not rated* (16%) [22]. Neesse et al. published similar observational data in the *P-CHEST* study [23]. Image quality was rated as *Excellent* (27%), *Mediocre* (44%) and *Poor* (29%) [23]. In the Ronaldson et al. study, expert reviewers graded 79% (*n* = 19) images as diagnostically adequate [5]. This involved a variety of settings including the back of ambulances, roadside and whilst in fixed- and rotary-wing aircraft. Roline et al. focussed purely on in-flight thoracic POCUS imaging [4]. The results of *n* = 81 saved images were reviewed by a recognised expert in POCUS who was blinded to flight-crew interpretations. They rated image quality as *Good* (54%) and *Poor* (44%) [4].

Snaith et al. compared the results of Extended Focused Assessment with Sonography in Trauma (eFAST) imaging conducted in an ED, versus a stationary ambulance, versus a moving ambulance [33]. A total of *n* = 36 examinations were performed in these settings by experienced emergency physicians or sonographers. When graded by an experienced clinical academic sonographer, no significant difference was observed between the quality of images produced. Although the mean time to conduct the eFAST examination was 20 s longer in the moving ambulance compared to the other two settings, this was not deemed statistically significant (*p* = 0.15). This study was a small feasibility study which the authors admit was likely underpowered.

The study by Lyon et al. differed in that it involved an airborne model consisting of an air-filled intravenous pressure bag placed inside another pressure bag to simulate the pleural interface of the lungs [24]. Air was injected between the bags to simulate pneumothorax. The images published in this paper show that the model produced a seemingly life-like depiction characteristic of an ultrasound image of the plural interface. Hence, despite this being a simulation study, it was deemed appropriate for inclusion in this review. A total of *n* = 16 M*-mode* images of the model pleura were obtained whilst in flight. These simulated no-pneumothorax (no air injected) and pneumothorax (air injected) in various flight configurations. Four emergency physicians experienced in the use of ultrasound to detect pneumothorax reviewed the captured images independently. They were blinded to the simulation and constituted the reference test on which to deduce the accuracy of *M-mode* imaging to detect pneumothorax in flight. They reported *M-mode* tracing during thoracic POCUS examination had a fine sawtooth wave pattern which was more pronounced in flight than on the ground [24]. However, this did not impede image interpretation. The authors concede that human tissue may have behaved differently.

Three studies involving *n* = 317 separate images reported the average time it took to complete the POCUS examination [6, 23, 25]. Most of these scans (60%, *n* = 190) were completed by nurses or paramedics. The mean time to conduct the P-CHEST assessment (including cardiac views) was two minutes and the time limit of five minutes was never exceeded [23]. Similarly, the average time to complete the entire eFAST examination (including cardiac and abdominal views) in a study by Yates et al. was also around two minutes [6]. Unexpectedly, overall on-scene time was reduced by an average of four minutes after the introduction of POCUS into this service [6]. This was attributed to the POCUS training program, and the result yielded by the eFAST examinations, improving decision making on rapid transportation. However, confounding due to the Hawthorne effect cannot be discounted. In the Scharonow et al. study, the time to complete a thoracic POCUS examination was approximately 30 s [25]. Regression analysis revealed that the use of ultrasound did not have a statistically significant impact on mission time [25]. The most common reason reported for poor image quality in studies was larger body habitus – a complication shared in all settings [5, 6, 22, 23, 35]. Authors also cited short flight times and packaging as barriers to image acquisition [2, 4].

### Synthesis of results: meta-analysis

Of the twelve studies yielded by the search strategy [1–6, 22–26, 33], four were excluded from meta-analysis [23, 25, 26, 33]. The Snaith et al. study was excluded as it did not report sufficient accuracy data [33]. The Neesse et al. and Scharonow et al. were excluded on account of there being no pneumothoraces present in the samples [23, 25]. Khalil et al. reported the number of thoracostomies performed [26]. As this outcome was not necessarily an indication of POCUS interpretation, this data was also excluded.

**Fig. 8:**
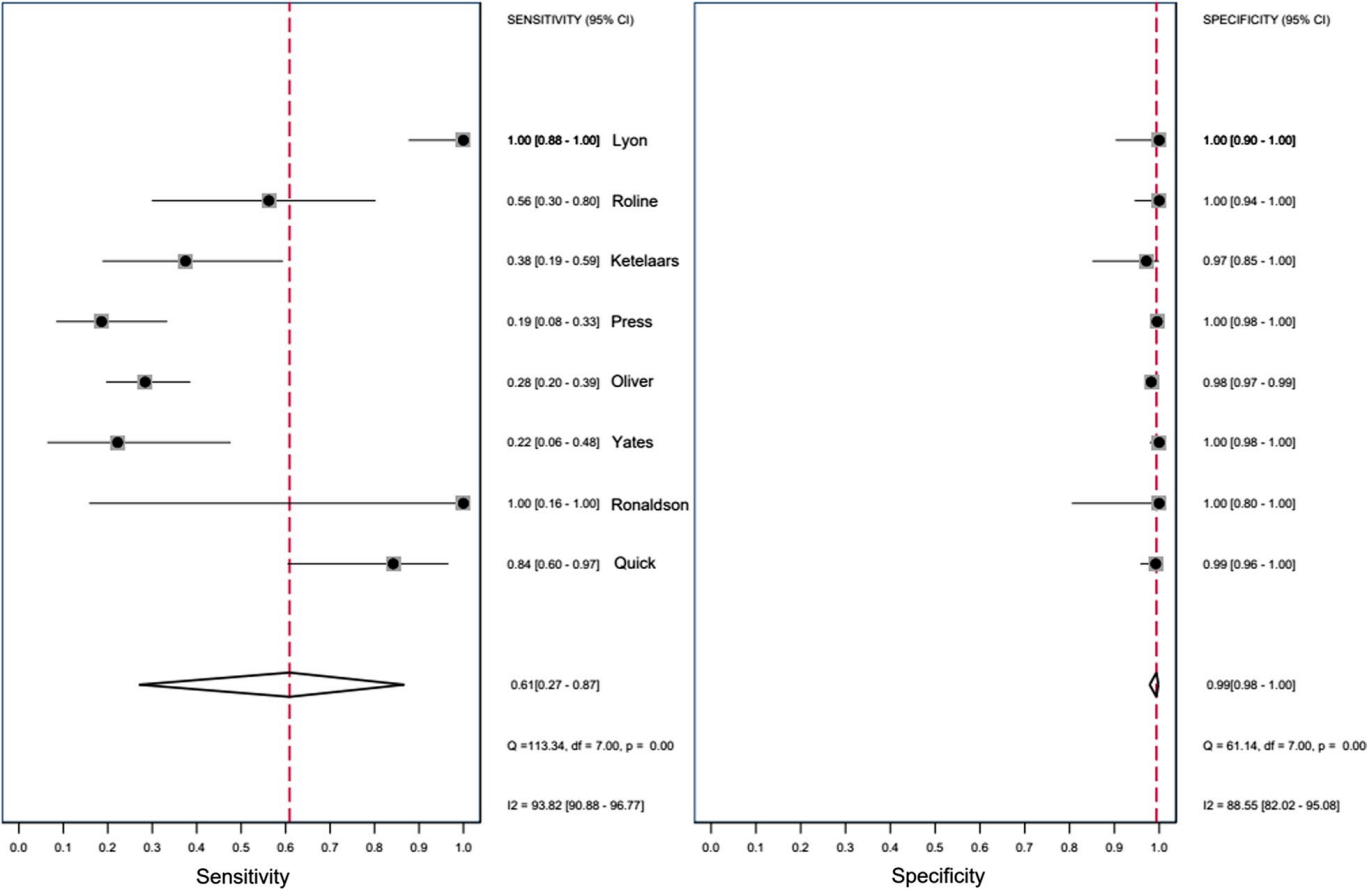
Meta-analysis: accuracy of POCUS for pneumothorax

The remaining eight studies reporting accuracy data involving *n* = 1,713 images were included in meta-analysis [1–6, 22, 24]. This yielded pooled sensitivity 61% (95% CI: 27–87%) and pooled specificity 99% (95% CI: 98–100%) (Fig. 8). There was a large degree of overall variance due to inter-study differences, thus indicating considerable heterogeneity: sensitivity *I*^2^ = 94% (95% CI: 91–97%) and specificity *I*^2^ = 89% (95% CI: 82–95%). The test for funnel plot asymmetry indicated no finding of publication bias (*p* = 0.14). The variations in methods of reporting outcomes rendered it impractical to conduct meaningful meta-analysis of the practicality results.

### Additional analysis

Studies involving multiple settings did not differentiate between in-flight and other settings when reporting accuracy. Hence, it was not possible to conduct subgroup analysis of results exclusively reporting in-flight image acquisition (nor any a posteriori identified subgroups) as intended due to a lack of data.

## Discussion

### Summary of evidence

This systematic review and meta-analysis quantified the sensitivity and specificity of thoracic POCUS for pneumothorax amongst HEMS providers. It also reports on the practicality of performing a thoracic POCUS examination in this setting. The included studies were all vulnerable to accusation of bias. Specificity results were unanimously precise and very high, whereas sensitivity results were imprecise and extremely variable. Meta-analysis results reflected this with low and imprecise pooled sensitivity blighted by considerable heterogeneity: (61% (95% CI: 27–87%; I2 = 94%). Pooled specificity results were precise and extremely high: 99% (95% CI: 98–100%; I2 = 89%). The highest or second highest categorisation of image quality was obtained in around half of patients.

### Accuracy

The extreme variability in the sensitivity results was expected as studies involved image acquisition and subjective interpretation in an extremely variable and unpredictable setting by operators of differing abilities... such is the nature of pre-hospital care. Included studies were also vulnerable to differences on account of variations in experimental methods between studies. Further investigation of the heterogeneity was not performed as the small numbers involved would not produce meaningful analysis [36]. These heterogeneity and bias vulnerabilities demand that interpretation and external application of these sensitivity findings to a specific setting or circumstance be done in an extremely cautious manner.

The pooled sensitivity result was much lower compared to previously published reviews involving the ED setting. The National Institute for Health and Care Excellence reported that thoracic POCUS in the ED for pneumothorax had pooled sensitivity 85% (95% CI: 68–95%) [37]. This was superior to X-ray [37]. Four other reviews reported similarly higher and more consistent sensitivity results and also corroborated the superiority of ED POCUS over X-ray [38–41]. This can be explained by the ED setting being more controlled, posing less environmental challenges. It may also be explained by differences in operator training and experience—the ED reviews differed in that they involved studies where operators were clinicians with previous experience in POCUS. However, a more recently published review in the ED setting by Netherton et al. reported a pooled sensitivity 69% (95% CI: 66–73%) [42]. For various reasons, this latter paper included different studies with lower sensitivities compared to the former reviews. These lower sensitivities may be partly explained by recent advances in CT imaging accuracy and higher instances of occult detection due to an increase in routine CT scanning.

Scrutiny of the forest plots published in these ED reviews revealed their sensitivity results were like the results in this paper in that they were variable, imprecise and had poor overlap of the 95% CIs [38–42]. Specificity results were similarly precise and near 100% [38–42]. Their pooled results also suffered with considerable heterogeneity [38–42]. Previously published reviews involving pre-hospital thoracic POCUS for pneumothorax are exclusively narrative in nature [43–48]. Authors cite a paucity in suitable evidence at the time of writing as the reason why they did not conduct quantitative analysis. However, they did unanimously conclude that pre-hospital ultrasound (including thoracic POCUS for pneumothorax) is feasible and useful, but only for some patients.

The extremely variable and seemingly unpredictable sensitivity results in this literature review renders thoracic POCUS an inappropriate tool to rule out pneumothorax in the HEMS setting. However, this is from purely a diagnostic and academic perspective, not necessarily a practical perspective considering sensitivity in terms of the clinical significance of the pneumothoraces. Two of the studies involving around a third (*n* = 679) of the total images also reported on only pneumothoraces requiring intervention [2, 6]. Yates et al. revealed a reduced prevalence (compared to all pneumothoraces) of 5% (95% CI: 3–10%) versus 9% (95% CI: 6–15%); but crucially, an increased sensitivity of 40% (95% CI: 12–74%) versus 22% (95% CI: 6–48%) [6]. The same trend was reported in the Press et al. study. They reported a reduced prevalence of 4% (95% CI: 2–6%) versus 9% (95% CI: 6–12%); and again, increased sensitivity: 50% (95% CI: 22–58%) versus 19% (95% CI: 9–34%) [2]. Three studies also reported on comparisons between pre-hospital thoracic POCUS versus in-hospital diagnosis (prior to CT) [2, 6, 22]. In the Yates et al. study, when the receiving trauma team’s assessment was used as the reference test, sensitivity increased to 67% (95% CI: 22–96%) versus 22% (95% CI: 6–48%) [6]. Similarly, the poor sensitivity rate in the Press et al. study was replicated in X-ray imaging [2]. Of the *n* = 35 false negative HEMS interpretations, *n* = 31 were also false-negative on X-ray. Sensitivity in the Ketelaars et al. study was also comparable to X-ray [22]. Of the *n* = 15 false negatives, *n* = 12 were also false negative on X-ray. Pneumothorax was only evident on CT in these cases, thus rendering their significance questionable [22].

The accuracy results of this literature review appear to present an overly pessimistic representation of sensitivity when considered in the context of clinical significance. Sensitivity improves when evaluating patient focussed outcomes rather than diagnostic ones. Although some pneumothoraces are missed, these are only apparent on CT (not clinically or on X-ray). For the missed cases that subsequently underwent an in-hospital intervention, the appropriateness of intervening in these cases in the HEMS setting is debatable.

The difficulty of conducting a clinical assessment (specifically auscultating) in the HEMS setting seemingly hinders diagnosis of pneumothorax [9, 10]. Combining difficulties in diagnosis with an imperative to treat this potentially life threatening condition may contribute towards performing unnecessary pre-hospital thoracostomies. A study reviewing *n* = 56 pre-hospital thoracostomies revealed around a quarter were unnecessary as no pneumothorax had been present [49]. Another study reported no evidence of pneumothorax in 79% (*n* = 15) of *n* = 19 cases where pre-hospital thoracostomies had been performed [50]. One HEMS service reported blindly performing thoracostomies on all pulseless trauma patients to relieve a potential tension pneumothorax [12]. They concluded this subsequently appeared unnecessary in 90% (*n* = 130) of cases.

It appears that thoracic POCUS can be used to mitigate against performing such unnecessary thoracic procedures. Lyon et al. reported a 21% decrease in chest decompressions performed following the introduction of thoracic POCUS into service [51]. Other authors also corroborate this hypothesis [6, 22, 51, 52]. It appears that usefulness is not limited to mitigating unnecessary interventions. Case reports also describe how thoracic POCUS enabled timely differential diagnosis and directed targeted treatments in rapidly deteriorating patients [6, 35]. Nevertheless, there appears no high-quality evidence reporting the usefulness of thoracic POCUS for pneumothorax. The evidence is limited to case reports demonstrating benefit for a small number of patients only. Crucially, there is no suggestion of performing a thoracic POCUS examination having a deleterious effect.

### Practicality

Despite the challenges of the pre-hospital and helicopter environment, results revealed that it is possible to obtain diagnostically adequate images in the HEMS setting. In general, it was reported that the highest or second highest categorisation of image quality was obtained in around half of patients. Unexpectedly, the greatest barrier to image acquisition was cited as body habitus, as opposed to one of the difficulties more commonly associated with the pre-hospital and helicopter environments. In-flight image acquisition was reportedly more difficult, but nevertheless possible.

The time it took clinicians to perform the examination was negligible. Regardless, prolonging on-scene times is arguably a moot point. Concerns around prolonging on-scene times leading to worse outcomes are usually attributable to the *Golden Hour* mantra [53]. This timeliness paradigm dictates that on-scene time should be minimised for the critically injured. This is so their needs can be met at hospital within an hour of injury [53]. The crux is that this relies on the presupposition that their immediate needs can only be met at an appropriate trauma hospital. The advent of more advanced pre-hospital diagnostic and interventional capabilities means this is no longer the case [54]. Besides, some patients will require significant intervention to avoid mortality much sooner [55].

Most of the images in the studies included in the literature review were conducted by previous novices to ultrasound. They demonstrated they were able to acquire and interpret thoracic POCUS images after undergoing only short training courses. Studies evaluating such curricula conclude that these programmes enable operators to competently acquire and interpret thoracic images to assess for pneumothorax [27, 29, 56, 57]. This may be explained by pre-hospital clinicians being already familiar with locating intercostal spaces due to familiarity with performing needle thoracostomies. In addition, unlike some other aspects of ultrasound, differentiating between normal and pathological findings involves assessment for several relatively easily distinguishable features.

### Limitations

The study selection process was conducted by one person, thus rendering it vulnerable to selection bias. This was mitigated to some extent by application of an objective selection criteria and transparent reporting of the reasons for not including papers in both qualitative and quantitative analysis. Results yielded only a small number of studies, and these included relatively few images. The predominantly high and unclear risk of bias associated with the included studies compromises the validity of the meta-analytical estimates.

Meta-analysis was blighted by apparent considerable heterogeneity indicated by an *I*^2^ value ≥ 75%. However, calculating separate *I*^2^ statistics for sensitivity and specificity fails to account for any correlation between the two [36]. This can result in an over-estimation of the degree of heterogeneity [36]. Comparisons with the visual assessment of forest plots revealed that the high level of heterogeneity in sensitivity is corroborated by little overlap in some of the relatively wide (imprecise) 95% CIs. Conversely, the high level of heterogeneity in specificity is contested by a consistent overlap in their narrow 95% CIs. Whilst heterogeneity exists in the specificity results, its magnitude remains debatable.

It was not possible to conduct analysis of exclusively in-flight image acquisition, nor any a posteriori identified sub-groups.

In the Ronaldson et al. study, practitioners correctly diagnosed a pneumothorax that was excluded from analysis as the image was deemed not diagnostically adequate by the reviewers (pneumothorax was confirmed using X-ray) [5]. This highlights the inability to account for other clinical variables associated with pneumothorax that may bias diagnosis.

The test used for funnel plot asymmetry has low power when data is heterogeneous [20]. The *Cochrane Handbook for Systematic Reviews of Diagnostic Test Accuracy* recommends caution when using it to assess for publication bias in this these cases [21]. Hence, there appeared no useful method of determining the risk of publication bias that would yields meaningful results. However, unlike in meta-analysis of interventional data, it appears unclear to what extent (if at all) the potential for publication bias compromises the validity of meta-analysis of diagnostic test accuracy results.

This review was performed according to *PRISMA-DTA* checklist with a prospectively submitted protocol and application of validated tools. However, some aspects such as assessment of bias and determining sources of heterogeneity were unavoidably subjective.

## Conclusions

It is possible to acquire diagnostically adequate thoracic POCUS images during HEMS missions. It also appears that novices to ultrasound can be taught to acquire and interpret images in this setting after relatively short training programmes. Specificity results are consistently very high and precise. Sensitivity appears imprecise and extremely unpredictable. This can be explained by differences in operator ability, settings, and the various environmental challenges associated with this area of practice. Sensitivity appears to increase when only clinically significant pneumothoraces are considered. The relevance of the false negatives in the HEMS setting is debatable. Irrespective, POCUS appears superior to auscultation with a conventional stethoscope when assessing for pneumothorax. It can appropriately alter treatment and triage decisions, but only for a small number of patients. This is predominantly on account of its apparent potential to reduce the number of unnecessary procedures. This hypothesis and the benefits this may yield requires further research. Randomised controlled methodologies reporting on patient focused outcomes are required. Reporting on mortality or morbidity may prove impractical. Future research may need to involve patient focussed surrogate outcomes such as numbers of clinically significant pneumothoraces detected, or the appropriateness of pre-hospital thoracic interventions performed or withheld. Crucially, it should account for potential confounding. In the meantime, thoracic POCUS appears to offer a more appropriate visual (rather than audible) alternative to auscultation for breath sounds when assessing for pneumothorax in the HEMS setting. It is imperative that users remain mindful that in the HEMS setting, environmental factors can compromise the high sensitivity (but not the specificity) previously reported in studies involving the ED setting.

## Data Availability

Not applicable.

## Appendices

### Appendix 1: Search terms

*Embase* was searched using the following *EMTREE (/exp)* and free text *(ab,ti)* terms: ('emergency care'/exp OR 'air medical transport'/exp OR 'helicopter'/exp OR pre-hospital:ab,ti OR helicopter:ab,ti OR 'in flight':ab,ti OR 'emergency medical services':ab,ti) AND ('echography'/exp OR 'ultrasound'/exp OR 'ultrasound scanner'/exp OR ultrasound:ab,ti OR sonography:ab,ti OR PoCUS:ab,ti) AND ('pneumothorax'/exp OR pneumothorax:ab,ti) AND ('diagnosis'/exp OR 'accuracy'/exp OR 'diagnostic accuracy'/exp OR 'sensitivity'/exp OR 'specificity'/exp OR diagnosis:ab,ti OR accuracy:ab,ti OR feasibility:ab,ti OR sensitivity:ab,ti OR specificity:ab,ti OR 'predictive value':ab,ti).

*PubMed* was searched using the following *MeSH (MeSH Terms)* and free text *(Title/Abstract)* terms: ("Emergency medical services"[MeSH Terms] OR "aircraft"[MeSH Terms] OR "air ambulances"[MeSH Terms] OR ("pre-hospital"[Title/Abstract] OR "helicopter"[Title/Abstract] OR "In-flight"[Title/Abstract] OR "Emergency medical services"[Title/Abstract])) AND ("Ultrasound"[Title/Abstract] OR "Sonography"[Title/Abstract] OR "POCUS"[Title/Abstract] OR "ultrasonography, doppler"[MeSH Terms] OR "ultrasonography"[MeSH Terms]) AND ("diagnosis"[Title/Abstract] OR "accuracy"[Title/Abstract] OR "feasibility"[Title/Abstract] OR "Sensitivity"[Title/Abstract] OR "Specificity"[Title/Abstract] OR "predictive value"[Title/Abstract] OR "diagnosis"[MeSH Terms] OR "diagnostic equipment"[MeSH Terms] OR "diagnostic tests, routine"[MeSH Terms] OR "sensitivity and specificity"[MeSH Terms] OR "predictive value of tests"[MeSH Terms]) AND ("pneumothorax"[MeSH Terms] OR "pneumothorax"[Title/Abstract]).

The *Cochrane Library* was searched using the *MeSH (MeSH descriptor)* and free text *(ti,ab,kw)* terms: ((pre-hospital):ti,ab,kw OR (helicopter):ti,ab,kw OR (In-flight):ti,ab,kw OR (“Emergency medical services”):ti,ab,kw OR MeSH descriptor: [Air Ambulances] explode all trees OR MeSH descriptor: [Aircraft] explode all trees OR MeSH descriptor: [Emergency Medical Services] explode all trees) AND ((Ultrasound):ti,ab,kw OR (Sonography):ti,ab,kw OR (POCUS):ti,ab,kw OR MeSH descriptor: [Ultrasonography, Doppler] explode all trees OR MeSH descriptor: [Ultrasonography] explode all trees) AND ((diagnosis):ti,ab,kw OR (accuracy):ti,ab,kw OR (feasibility):ti,ab,kw OR (Sensitivity):ti,ab,kw OR (Specificity):ti,ab,kw OR MeSH descriptor: [Diagnostic Equipment] explode all trees OR MeSH descriptor: [Diagnostic Tests, Routine] explode all trees OR MeSH descriptor: [Sensitivity and Specificity] explode all trees OR MeSH descriptor: [Predictive Value of Tests] explode all trees) AND ((pneumothorax):ti,ab,kw OR MeSH descriptor: [Pneumothorax] explode all trees).

### Appendix 2: Risk of bias and applicability

Risk of bias and applicability assessments were done across four domains:

1. Patient selection
2. Index test
3. Reference standard
4. Flow and timing

Each domain involved signalling questions relating to the potential for bias or an applicability concern. If there was sufficient information in the publication to determine the apparent risk or concern, answers were recorded as *Yes* or *No*. When there was insufficient detail, the answer was recorded as *Unclear*. The standard QUADAS-2 signalling questions were tailored to include whether authors accounted for potential bias due to inter-reviewer reliability. An overall subjective assessment was then made as to whether the collective answers to these signalling questions constituted a risk of bias, or an applicability concern, in each domain. These assessments were then inputted into the QUADAS-2 assessment tool embedded in the Review Manager software (Version 5.4.1. The Nordic Cochrane Centre, The Cochrane Collaboration, Copenhagen, 2020).

These were guided subjective evaluations. Hence, there are methodological flaws in making these assessments and assigning weights to the responses in the respective domain evaluations. Attempting to combine them to reach a definitive overall objective assessment of quality across studies is similarly flawed. Therefore, results are presented graphically and as descriptive qualitative assessments. This avoids unjustified importance being afforded to any item or domain. It also ensures transparency of the assessments.

Ketelaars et al. only reported on *n* = 59 out of a possible *n* = 326 patients as the authors did not have access to the reference test results for the other *n* = 267 cases. Similarly, Oliver et al. reported on *n* = 331 cases having lost *n* = 2,046 patients to follow-up. This was on account of these patients not being taken to the hospital that was participating in the study. They also excluded another *n* = 50 patients as they did not undergo a CT scan. Ronaldson et al. randomly selected *n* = 12 from a potential *n* = 29 eligible patients who met the inclusion criteria. These quasi-convenience sampling methods resulted in an assessment of a high risk of bias in *patient selection*. Only Quick et al. accounted for inter-reviewer reliability in the pre-hospital setting. The remaining studies were assessed as involving an unclear risk bias in the *index test* domain on account of them not reporting on the potential for varying degrees of pre-hospital image interpretation abilities. Three studies used expert interpretation of the original captured images as the reference standard. As this appeared to assess pre-hospital versus in-hospital inter-reviewer reliability (rather than reporting diagnostic accuracy), it was evaluated as incurring a high risk of bias in the *reference standard* domain.

Four studies did not report whether the reference standard results were interpreted without knowledge of the index test. This resulted in an assessment of an unclear risk of bias. A small number of patients in three studies did not undergo the intended primary reference test (CT). This was because of their deteriorating clinical condition and obvious indication of pneumothorax by other means. Chest X-ray, definitive clinical signs of a pneumothorax, or the trauma surgeon’s operative report, were used as the reference test in these cases. The degree of the risk of bias this incurred in this context was difficult to determine. This was recorded as an unclear risk of bias assessment in the *flow and timing* domain. Nearly all studies raised low concerns about applicability. Only the Lyon et al. study raised high concerns due to the use of a model to simulate the plural interface. This model simulated the plural interface but did not simulate human tissue and structures which may have produced different results in the airborne environment.

## Abbreviations

CI: Confidence interval
CT: Computed tomography
ED: Emergency department
eFAST: Extended Focused Assessment with Sonography in Trauma
HEMS: Helicopter emergency medical services
M-mode: Motion mode
NPV: Negative predictive value
PROSPERO: International Prospective Register of Systematic Reviews
POCUS: Point of care ultrasound
PPV: Positive predictive value
PRISMA-DTA: Preferred reporting items for systematic reviews and meta-analysis for diagnostic test accuracy
QUADAS-2: Quality Assessment of Diagnostic Accuracy Studies

## References

[1] Oliver P, Bannister P, Bootland D, Lyon RM (2020). Diagnostic performance of prehospital ultrasound diagnosis for traumatic pneumothorax by a UK Helicopter Emergency Medical Service. EurJ Emerg Med..

[2] Press GM, Miller SK, Hassan IA, Alade KH, Camp E, Del Junco D (2014). Prospective evaluation of prehospital trauma ultrasound during aeromedical transport. J Emerg Med..

[3] Quick JA, Uhlich RM, Ahmad S, Barnes SL, Coughenour JP (2016). In-flight ultrasound identification of pneumothorax. Emerg Radiol..

[4] Roline CE, Heegaard WG, Moore JC, Joing SA, Hildebrandt DA, Biros MH (2013). Feasibility of bedside thoracic ultrasound in the helicopter emergency medical services setting. Air Med J..

[5] Ronaldson J, Moultrie CEJ, Corfield AR, McElhinney E. Can non-physician advanced retrieval practitioners (ARP) acquire and interpret diagnostic views of the lungs with sufficient quality to aid in the diagnosis of pneumothorax in the pre-hospital and retrieval environment. Scand J Trauma Resusc Emerg Med. 2020; 28(Art No.102 (2020)). 10.1186/s13049-020-00797-8#. 10.1186/s13049-020-00797-8 PMC7565770 33066800

[6] Yates JG, Baylous D (2017). Aeromedical ultrasound: the evaluation of point-of-care ultrasound during helicopter transport. Air Med J..

[7] Leigh-Smith S, Harris T (2005). Tension pneumothorax—time for a re-think. Emerg Med J..

[8] Knotts D, Arthur AO, Holder P, Herrington T, Thomas SH (2013). Pneumothorax volume expansion in helicopter emergency medical services transport. Air Med J..

[9] Brown LH, Gough JE, Bryan-Berg DM, Hunt RC (1997). Assessment of breath sounds during ambulance transport. Ann Emerg Med..

[10] Hunt R, Bryan D, Brinkley V, Whitley T, Benson N (1991). Inability to assess breath sounds during air medical transport by helicopter. JAMA..

[11] Hernandez MC, El Khatib M, Prokop L, Zielinski MD, Aho JM (2018). Complications in tube thoracostomy: systematic review and meta-analysis. J Trauma Acute Care Surg..

[12] Peters J, Ketelaars R, van Wageningen B, Biert J, Hoogerwerf N (2017). Prehospital thoracostomy in patients with traumatic circulatory arrest: results from a physician-staffed helicopter emergency medical service. Eur J Emerg Med..

[13] Husain LF, Hagopian L, Wayman D, Baker WE, Carmody KA (2012). Sonographic diagnosis of pneumothorax. J Emerg Trauma Shock..

[14] Bowra J, Loubani O, Atkinson P, Atkinson P, Bowra J, Harris T, Jarman B, Lewis D (2019). Chapter 3: The chest. Point of care ultrasound for emergency medicine and resuscitation.

[15] Yue Lee FC, Jenssen C, Dietrich CF (2018). A common misunderstanding in lung ultrasound: the comet tail artefact. Med Ultrason..

[16] Resuscitation Council UK. 2021 Resuscitation Guidelines. 2021. Available from: https://www.resus.org.uk/library/2021-resuscitation-guidelines.

[17] Lott C, Truhlář A, Alfonzo A, Barelli A, González-Salvado V, Hinkelbein J (2021). European Resuscitation Council Guidelines 2021: Cardiac arrest in special circumstances. Resuscitation.

[18] McInnes MDF, Moher D, Thombs BD, McGrath TA, Bossuyt PM, The PRISMA-DTA Group (2018). Preferred reporting items for a systematic review and meta-analysis of diagnostic test accuracy studies: The PRISMA-DTA statement. JAMA. 2018; 319(4): 388–396. 10.1001/jama.2017.19163. 10.1001/jama.2017.19163 29362800

[19] Whiting PF, Rutjes AWS, Westwood ME, Mallett S, Deeks JJ, Reitsma JB (2011). QUADAS-2: a revised tool for the quality assessment of diagnostic accuracy studies. Ann Intern Med..

[20] Deeks JJ, Macaskill P, Irwig L (2005). The performance of tests of publication bias and other sample size effects in systematic reviews of diagnostic test accuracy was assessed. J Clin Epidemiol..

[21] Macaskill P, Gatsonis C, Deeks J, Harbord R, Takwoingi Y. Chapter 10: Analysing and presenting results. In: Deeks JJ, Bossuyt PM, Gatsonis C (editors), Cochrane Handbook for Systematic Reviews of Diagnostic Test Accuracy. 2010.

[22] Ketelaars R, Hoogerwerf N, Scheffer GJ (2013). Prehospital chest ultrasound by a Dutch helicopter emergency medical service. J Emerg Med..

[23] Neesse A, Jerrentrup A, Hoffmann S, Sattler A, Görg C, Kill C (2012). Prehospital chest emergency sonography trial in Germany: a prospective study. Eur J Emerg Med..

[24] Lyon M, Shiver SA, Walton P (2012). M-mode ultrasound for the detection of pneumothorax during helicopter transport. Am J Emerg Med..

[25] Scharonow M, Weilbach C. Prehospital point-of-care emergency ultrasound: a cohort study. Scand J Trauma Resusc Emerg Med. 2018; 26(Art No.49 (2018)). 10.1186/s13049-018-0519-9 PMC6006664 29914554

[26] Khalil PA, Merelman A, Riccio J, Peterson J, Shelton R, Meyers J (2020). Randomized controlled trial of point-of-care ultrasound education for the recognition of tension pneumothorax by paramedics in prehospital simulation. Prehosp Disaster Med..

[27] Chin EJ, Chan CH, Mortazavi R, Anderson CL, Kahn CA, Summers S (2013). A pilot study examining the viability of a prehospital assessment with ultrasound for emergencies (PAUSE) protocol. J Emerg Med..

[28] Lyon M, Walton P, Bhalla V, Shiver SA (2012). Ultrasound detection of the sliding lung sign by prehospital critical care providers. Am J Emerg Med..

[29] Bhat SR, Johnson DA, Pierog JE, Zaia BE, Williams SR, Gharahbaghian L (2015). Prehospital evaluation of effusion, pneumotorax, and standstill (PEEPS): point-of-care ultrasound in emergency medical services. West J Emerg Med..

[30] Price DD, Wilson SR, Murphy TG (2000). Trauma ultrasound feasibility during helicopter transport. Air Med J..

[31] Brun P, Bessereau J, Levy D, Billeres X, Fournier N, Kerbaul F (2014). Prehospital ultrasound thoracic examination to improve decision making triage and care in blunt trauma. Am J Emerg Med.

[32] Becker TK, Martin-Gill C, Callaway CW, Guyette FX, Schott C (2018). Feasibility of paramedic performed prehospital lung ultrasound in medical patients with respiratory distress. Prehosp Emerg Care..

[33] Snaith B, Hardy M, Walker A (2011). Emergency ultrasound in the prehospital setting: the impact of environment on examination outcomes. Emerg Med J..

[34] Brun P, Bessereau J, Chenaitia H, Pradel A, Deniel C, Garbaye G (2014). Stay and play eFAST or scoop and run eFAST? That is the question. Am J Emerg Med..

[35] Madill JJ (2010). In-flight thoracic ultrasound detection of pneumothorax in combat. J Emerg Med..

[36] Bossuyt P, Davenport C, Deeks J, Hyde C, Leeflang M, Scholten R. Chapter 11: Interpreting results and drawing conclusions. In: Deeks JJ, Bossuyt PM, Gatsonis C (editors), Cochrane handbook for systematic reviews of diagnostic test accuracy. 2013.

[37] National Institute for Health and Care Excellence. Major trauma: assessment and initial management (NICE Guideline NG39)—methods, evidence and recommendations. 2016. 26913320

[38] Alrajhi K, Woo MY, Vaillancourt C (2012). Test characteristics of ultrasonography for the detection of pneumothorax: a systematic review and meta-analysis. Chest..

[39] Staub LJ, Biscaro RRM, Kaszubowski E, Maurici R (2018). Chest ultrasonography for the emergency diagnosis of traumatic pneumothorax and haemothorax: A systematic review and meta-analysis. Injury..

[40] Alrajab S, Youssef AM, Akkus NI, Caldito G. Pleural ultrasonography versus chest radiography for the diagnosis of pneumothorax: review of the literature and meta-analysis. Crit Care. 2013; 17(Art No.R208 (2013)): R208. 10.1186/cc13016. 10.1186/cc13016 PMC4057340 24060427

[41] Ding W, Shen Y, Yang J, He X, Zhang M (2011). Diagnosis of pneumothorax by radiography and ultrasonography: a meta-analysis. Chest..

[42] Netherton S, Milenkovic V, Taylor M, Davis PJ (2019). Diagnostic accuracy of eFAST in the trauma patient: a systematic review and meta-analysis. CJEM..

[43] Bøtker MT, Jacobsen L, Rudolph SS, Knudsen L. The role of point of care ultrasound in prehospital critical care: a systematic review. Scand J Trauma Resusc Emerg Med. 2018; 26(Art No.51 (2018)). 10.1186/s13049-018-0518-x PMC6019293 29940990

[44] Ketelaars R, Reijnders G, van Geffen G, Scheffer GJ, Hoogerwerf N. ABCDE of prehospital ultrasonography: a narrative review. Crit Ultrasound J. 2018; 10(Art No.17 (2018)): 17. 10.1186/s13089-018-0099-y PMC6081492 30088160

[45] Rudolph SS, Sørensen MK, Svane C, Hesselfeldt R, Steinmetz J (2014). Effect of prehospital ultrasound on clinical outcomes of non-trauma patients—a systematic review. Resuscitation..

[46] O'Dochartaigh D, Douma M (2015). Prehospital ultrasound of the abdomen and thorax changes trauma patient management: a systematic review. Injury..

[47] Jørgensen H, Jensen CH, Dirks J (2010). Does prehospital ultrasound improve treatment of the trauma patient? A systematic review. Eur J Emerg Med..

[48] Corcoran F, Bystrzycki A, Masud S, Mazur SM, Wise D, Harris T (2016). Ultrasound in pre-hospital trauma care. Trauma..

[49] Blaivas M (2010). Inadequate needle thoracostomy rate in the prehospital setting for presumed pneumothorax: an ultrasound study. J Ultrasound Med..

[50] Cullinane DC, Morris JA, Bass JG, Rutherford EJ (2001). Needle thoracostomy may not be indicated in the trauma patient. Injury..

[51] Lyon R, Weaver A, Wise D, Davies G, Lockey D (2012). Is there a role for pre-hospital chest ultrasound in trauma patients. Resuscitation..

[52] Jones RA, Tabbut M, Emerman C, Stout S (2014). The effect of thoracic ultrasound for the detection of pneumothorax on medical decision making in trauma patients in the out-of-hospital setting. Ann Emerg Med..

[53] Rogers FB, Rittenhouse KJ, Gross BW (2015). The golden hour in trauma: dogma or medical folklore. Injury..

[54] Maddock A, Corfield AR, Donald MJ, Lyon RM, Sinclair N, Fitzpatrick D (2020). Prehospital critical care is associated with increased survival in adult trauma patients in Scotland. Emerg Med J..

[55] Alarhayem AQ, Myers JG, Dent D, Liao L, Muir M, Mueller D (2016). Time is the enemy: Mortality in trauma patients with hemorrhage from torso injury occurs long before the "golden hour". Am J Surg..

[56] Mason R, Latimer A, Vrablik M, Utarnachitt R (2019). Teaching flight nurses ultrasonographic evaluation of esophageal intubation and pneumothorax. Air Med J..

[57] Noble VE, Lamhaut L, Capp R, Bosson N, Liteplo A, Marx J (2009). Evaluation of a thoracic ultrasound training module for the detection of pneumothorax and pulmonary edema by prehospital physician care providers. BMC Med Educ..

